# Regulation of cardiac microRNAs by serum response factor

**DOI:** 10.1186/1423-0127-18-15

**Published:** 2011-02-08

**Authors:** Xiaomin Zhang, Gohar Azhar, Scott A Helms, Jeanne Y Wei

**Affiliations:** 1 Donald W. Reynolds Department of Geriatrics, The University of Arkansas for Medical Sciences and Geriatric Research, Education and Clinical Center, Central Arkansas Veterans Healthcare System, Little Rock, AR 72205, USA

## Abstract

Serum response factor (SRF) regulates certain microRNAs that play a role in cardiac and skeletal muscle development. However, the role of SRF in the regulation of microRNA expression and microRNA biogenesis in cardiac hypertrophy has not been well established. In this report, we employed two distinct transgenic mouse models to study the impact of SRF on cardiac microRNA expression and microRNA biogenesis. Cardiac-specific overexpression of SRF (SRF-Tg) led to altered expression of a number of microRNAs. Interestingly, downregulation of miR-1, miR-133a and upregulation of miR-21 occurred by 7 days of age in these mice, long before the onset of cardiac hypertrophy, suggesting that SRF overexpression impacted the expression of microRNAs which contribute to cardiac hypertrophy. Reducing cardiac SRF level using the antisense-SRF transgenic approach (Anti-SRF-Tg) resulted in the expression of miR-1, miR-133a and miR-21 in the opposite direction. Furthermore, we observed that SRF regulates microRNA biogenesis, specifically the transcription of pri-microRNA, thereby affecting the mature microRNA level. The mir-21 promoter sequence is conserved among mouse, rat and human; one SRF binding site was found to be in the mir-21 proximal promoter region of all three species. The mir-21 gene is regulated by SRF and its cofactors, including myocardin and p49/Strap. Our study demonstrates that the downregulation of miR-1, miR-133a, and upregulation of miR-21 can be reversed by one single upstream regulator, SRF. These results may help to develop novel therapeutic interventions targeting microRNA biogenesis.

## Background

MicroRNAs (miRNAs) are short (20 to 23-nucleotide), endogenous, single-stranded RNA molecules that regulate gene expression by hybridization to messenger RNAs (mRNAs) with the consequence of mRNA degradation or translational inhibition of targeted transcripts. Genes that encode for miRNA are transcribed by either RNA polymerase II or RNA polymerase III into primary miRNA (pri-miRNA) transcripts, which are then cleaved by the nuclear microprocessor complex formed by the RNase III enzyme Drosha (RNASEN) and the DGCR8 (DiGeorge critical region 8) protein. The RNase III Dicer cleaves off the loop of the pre-miRNA to generate a roughly 22-nucleotide miRNA duplex [1].

Although insights into the regulatory function of miRNAs toward their mRNA targets are beginning to emerge, less is known about the regulation of miRNA gene expression and miRNA biogenesis [1]. For instance, it has been shown that miRNAs participate in the control of cardiac development, and a number of miRNAs play a role in cardiac hypertrophy [2-5]. In addition, serum response factor (SRF), an important transcription factor, participates in the regulation of several cardiac enriched miRNAs, including mir-1 and mir-133a [4,6]. However, it is unclear at what specific stage SRF regulates the biogenesis of miRNA.

SRF is a member of the MADS (MCM1, agamous, deficiens, SRF) family of transcriptional activators that has been implicated in the regulation of a number of genes that are important in cell proliferation and differentiation. SRF regulates its target genes by binding to the serum response element (SRE), which contains a consensus CC(A/T_6_)GG (CArG) motif [7-9]. This cognate response site of SRF is found in the promoter region of certain immediate-early genes and many muscle-specific genes [9-11]. Two CArG-like elements have been found in the promoter of mir-1-1 and mir-1-2 genes [4]. The level of SRF expression apparently changes during development and adult aging [12]. Therefore, it is plausible that SRF may impact not only cardiac muscle genes but also certain miRNA genes throughout one's lifespan.

In the present study, we utilized SRF transgenic mouse models to study the impact of serum response factor (SRF) on cardiac miRNA expression, and miRNA biogenesis. We observed that approximately 1/10 of the recently identified 578 miRNAs are highly expressed in the mouse heart; SRF overexpression in the mouse heart resulted in altered expression of a number of miRNAs, including the down-regulation of mir-1 and mir-133a, and up-regulation of mir-21, which are usually dysregulated in cardiac hypertrophy and congestive heart failure [3,13-16]. When the mouse cardiac SRF level was reduced using the antisense-SRF transgenic approach, we observed an increase in expression of miR-1 and miR-133a miRNA, and a decrease in expression of miR-21. In addition, we observed that SRF executes its regulatory role on miRNA expression through controlling the transcription of the pri-miRNA, the first step of miRNA biogenesis. Our findings demonstrate for the first time that it is possible to regulate at the same time the expression of three miRNAs, miR-1, miR-133a and miR-21, through targeting the components of SRF-mediated signaling pathway.

## Materials and methods

### Two transgenic mouse models

**Figure 1:**
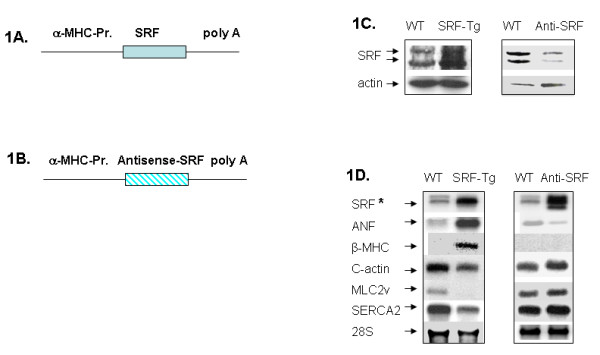
**Two models of transgenic mice**. **1A and 1B**: Scheme of DNA constructs for the generation of two distinct transgenic mouse models, in which the α-MHC-Promoter (α-MHC-Pr.) drives the transgene expression in the postnatal heart. 1A. DNA construct for the generation of cardiac-specific overexpression of SRF transgenic (SRF-Tg) mice. 1B. DNA construct for the generation of cardiac-specific antisense-SRF transgenic (Anti-SRF-Tg) mice. **1C**. Western blot revealed increased SRF protein in SRF-Tg versus wild-type (WT) mice, and decreased SRF protein level in Anti-SRF-Tg versus WT mice. **1D**. Northern blot analysis of cardiac gene expression in wild-type (WT) versus SRF-Tg, as well as WT versus Anti-SRF-Tg (Anti-SRF) mice. The SRF transcript (SRF*) was detected by a ^32^P-labelled double-strand SRF cDNA fragment, which hybridizes with both sense-SRF transcript and antisense-SRF transcript, respectively. The probes for ANF, β-MHC, cardiac actin, MLC2v and SERCA2 are ^32^P-labeled oligonucleotides.

The wild-type FVB mice and the transgenic mice on FVB background ranged from age at 7 days to 6 months. To study the effect of SRF level on cardiac gene expression and function in the mouse heart, we employed the cardiac-specific transgenic approach to increase the SRF protein level in one mouse model, and to reduce it in another mouse model. Figure 1A shows that in SRF overexpression transgenic mice (SRF-Tg), the α-MHC promoter drives the expression of a full-length SRF cDNA, which causes the cardiac-specific elevation of SRF by approximately 40%; the cardiac phenotype of this model has been previously reported [12]. Figure 1B shows that in antisense-SRF transgenic mice (Anti-SRF-Tg), the α-MHC promoter drives the expression of the full-length SRF cDNA in the antisense orientation. The transgenic overexpression of antisense-SRF RNA sequence interferes with the endogenous SRF mRNA and consequently reduces SRF protein level in the mouse heart (Figure 1C)[17]. The mRNA expression levels of 6 cardiac genes were shown in Figure 1D.

After euthanasia, the hearts were removed from mice and subjected to standard RNA isolation and histological procedures. For each time point, there were three independent biological replicates. The studies were conducted with Institutional Review Board approval and in accordance with the NIH Guiding Principles for Research Involving Animals.

### Total RNA isolation

All RNA samples were first isolated from the mouse cardiac ventricles using UltraSpec RNA Isolation Reagent as previously described [12]. To clean mouse DNA contamination and enrich the small RNA fraction, the total RNA samples were purified using miRNeasy Mini kit (Qiagen) and RNase-free DNase I according to the manufacture's instruction manual.

### Northern Blotting and Western Blotting

The Northern blotting was carried out as previously described [18,19]. Briefly, a ^32^P-labelled double-stranded SRF cDNA fragment from plasmid pCGNSRF was used as the probe for the detection of SRF mRNA in wild type mouse and SRF-Tg heart, as well as the antisense-SRF transcripts in Anti-SRF-Tg heart, respectively. The ANF, β-MHC, cardiac actin, MLC2v and SERCA2 transcripts were detected by ^32^P-labeled oligonucleotides. The sequences of the oligonucleotide probes were as follows: ANF, 5'-ccggaagctgttgcagcctagtccactctgggctccaatcctgtcaatcctacccccgaagcagctgga-3'; β-MHC: 5'-gagggcttcacgggcacccttagagctgggtagcacaagatctactcctcattcaggcc-3'. Ventricular myosin light chain-2 (MLC2v) isoform: 5'-cacagccctgggatggagagtgggctgtgggtcacctgaggctgtggttcag-3'. Cardiac α-actin: 5'-agggggctcagaggattccaagaagcacaatacggtcatcctgaatataaggtaggctaa-3'; sarcoplasmic reticulum Ca^2+^-ATPase (SERCA2), 5'-tcagtcatgcagagggctggtagatgtgttgctaacaacgcacatgcacgcacccgaaca-3'.

The Western blotting was performed as previously described [18,19]. Briefly, 50 ug of protein was separated by SDS-PAGE on a polyacrylamide gel and transferred to nitrocellulose. The membrane was blocked for 2 h at room temperature in 5% nonfat milk in Tris 20 mM, sodium chloride 137 mM, 0.1% Tween-20, pH 7.6 (TBS-T) and then incubated for 2 h at room temperature with SRF antibody followed by incubation for 1 h with horseradish peroxidase conjugated secondary antibody. Immunoreactive bands were visualized by chemiluminescence (ECL, Amersham International). The antibodies were purchased from Sigma, and Santa Cruz Biotechnology.

### MicroRNA arrays

The ventricular tissue samples used for the microRNA array analysis were obtained from 6-month-old wild-type mice and age-matched SRF-Tg mice. The RNA sample isolation and microRNA array were performed in triplets for both wild-type and transgenic mice. A total of 6 RNA samples of ventricular tissue were shipped on dry ice to Exiqon, Inc., which provided the service for RNA quality verification and microRNA array hybridization as well as comprehensive statistical analysis. The microarray data have been deposited in the NCBI Gene Expression Omnibus (GEO) database (http://www.ncbi.nlm.nih.gov/geo/) under accession no. GSE23044. Briefly, each pair of wild-type and transgenic heart samples were labeled with Hy3 and Hy5 fluorescent dyes respectively and hybridized to a miRCURY LNA™ mouse microRNA Array (version 10.0), which held 578 mature microRNA probes, as well as perfectly matched and mismatched probes for quality control. After signal amplification, the background was subtracted and normalized using LOWESS (Locally Weighted Scatter plot Smoothing) regression algorithm. This within-slide normalization was performed to minimize differences between the colors in an intensity-dependent manner. The array output was received in Excel spreadsheets containing the normalized microRNA expression profiles in each heart sample, the expression comparison between transgenic versus wild-type heart samples and "Expression Matrix" containing normalized Hy3/Hy5 ratios (log2 transformed) from all hybridizations. The list was sorted based on the most variant expressed miRNAs comparing the two sample types. 20 of 578 miRNA passed the filtering criteria with average "log-Median-Ratio" >0.50, which represents at least >1-fold change in microRNA expression.

### Real-time PCR quantitation of pri-miRNAs and mature RNAs

#### Detection of pri-miRNAs

The primers for the detection of pri-miRNAs were designed using PRISM Primer Express 3.0 software (Applied Biosystems), and synthesized at Integrated DNA Technologies Inc. The first-strand cDNA synthesis was carried out using random hexamer primer, and the PCR was performed using the following primers: pri-mir-1-1 forward: 5'-ccaagtgtgcatgtgtgagag-3', pri-mir-1-1 reverse: 5'-atgtctgacgagcacttccac-3'. pri-mir-1-2 forward: 5'-accacaagcagaagtggcatt-3', pri-mir-1-2 reverse: 5'-tggaagtcatcctcctggaaa-3'. pri-mir-21 forward: 5'-ccagagatgtttgctttgctt-3', pri-mir-21 reverse: 5'-tgccatgagattcaacagtca-3'. pri-mir-133a1 forward 5'-cactgatgtgagctgcaagaa-3', pri-mir-133a1 reverse 5'-tccaaataaggttgacagttgct-3'. pri-mir-133a2 forward 5'-caagaactgcttttccccttc-3, pri-mir-133a2 reverse 5'-tttcttggatctgaccattgc-3'. pri-mir199a1 forward 5'-ctgaggaactgaacagccatc-3', pri-mir199a1 reverse: 5'-gtctggaagttcccactgttg-3'. pri-mir381 forward: 5'-tggtacttaaagcgaggttgc-3', pri-mir381 reverse: 5'-ggtcatgcacacacataccac-3'. pri-mir499 forward: 5'-gcatgtgaacatcacagcaag-3', pri-mir499 reverse: 5'-ccaaacaccacctaagtcttc-3'.

#### Detection of other genes

Myocardin, forward primer 5'-ccaatcaattcccaggaaagc-3' and reverse primer 5'-tgggaccatttgggagtcaa-3. Zipzap, forward primer 5'-tcttgactgaactctggcactca-3' and reverse primer 5'-ctctgttctgtggccttggaa-3'. P49/Strap, forward primer: 5'-agaagtgaagcgaatccgagtt-3 and reverse primer 5'-gtctgccgacactcctgaca-3'. Atrial natriuretic factor (ANF) forward primer 5'-tgggacccctccgatagatc and reverse primer 5'-agcgagcagagccctcagt-3'.

The U5G small nuclear RNA (U5G) gene was used as an internal control: U5G forward: 5'-aaagatttccgtggagaggaa-3', U5G reverse: 5'-ccaagacaaggcctcaaaaa-3'. The PCR amplification was performed in a 7900HT Fast Sequence Detector System (Applied Biosystems) with following program: Cycle 1, 95°C for 10 minutes. Cycle 2, 40 cycles of 95°C for 15 seconds, 60°C for 60 seconds. Cycle 3, 95°C for 15 seconds, 60°C for 15 seconds, 95°C for 15 seconds. CT values were automatically obtained. Relative expression values were obtained by normalizing CT values of the miRNA genes in comparison with CT values of the U5G gene, using the CT method [20,21].

#### Detection of mature miRNAs

To detect each mature miRNA, the first-strand cDNA synthesis was carried out with a miRNA-specific primer, and then PCR was carried out with a miRNA-specific LNA-PCR primer and a universal PCR primer. The primers for miR-1, miR-21, miR-133a, miR-199a, miR-381 and miR-499-5p, 5S RNA reference primers, the cDNA synthesis kit and real-time PCR reagents were obtained from Exiqon Inc. The PCR amplification was performed under the conditions as mentioned above.

### Plasmid constructs

The mmu-mir-21 gene was amplified with forward primer 5'-aatggatccaaacagctttctttcctagaattgg-3' and reverse primer 5'-aagtctcacaagacataaggaccacaagctt tta-3' with PCR. The PCR fragment was then ligated into BamHI and HindIII digested pSilencer 4.1 vector. A DNA fragment containing mmu-mir-21 proximal promoter region (-3 bp to -589 bp) was amplified with forward primer 5'-tactcgagtaaagggtacaggaagtaagggt-3' and reverse primer 5'-atcaagctt ttctgagaagtcccacatttat-3', and subsequently cloned into pGL3 vector. All the DNA constructs were confirmed by sequencing analysis. Other plasmid constructs that were used in the study have been previously described, which include pGL3-ANF promoter vector, pGL3-cardiac actin promoter vector, pAdTrack-CMV, pAd-CMV-wtSRF, pcDNA3-49/Strap, pcDNA3-Zipzap, pcDNA3-SRF-delta-3 Nkx2.5 and GATA-4 [12,19,22,23]. The wild-type myocardin plasmid is a generous gift of Dr. E. Olson [24].

### Transfection Assays

Transient transfections were carried out with the Lipofectamine 2000 reagents (Invirogen) as previously described [22,25]. At 4 h after the transfection was initiated, the NIH3T3 cells were placed in Dulbecco's modified Eagle's medium with 10% fetal calf serum and incubated overnight. The cells were then cultured in Dulbecco's modified Eagle's medium with 0.1% fetal calf serum for another 24 h and then placed in Dulbecco's modified Eagle's medium with 20% fetal calf serum for an additional 3.5 h. Firefly luciferase activity was measured as relative light units. To control for variability, the number of relative light units from individual transfection experiments was normalized by measuring Renilla luciferase activity expressed from a cytomegalovirus promoter-driven vector in the same samples. Individual transfection experiments were carried out in triplicate, and the results were reported as mean firefly luciferase/Renilla luciferase activity (mean ± S.D.) from representative experiments.

### Statistical analysis

Data are given as mean values ± SD, with *n *denoting the number of experiments unless otherwise indicated. A list of differentially expressed microRNAs were identified using a t-test with a combination of cut off p-value (p < 0.05) and at least 1-fold change.

## Results

### 1. The microRNA expression in wild-type mouse hearts

**Figure 2:**
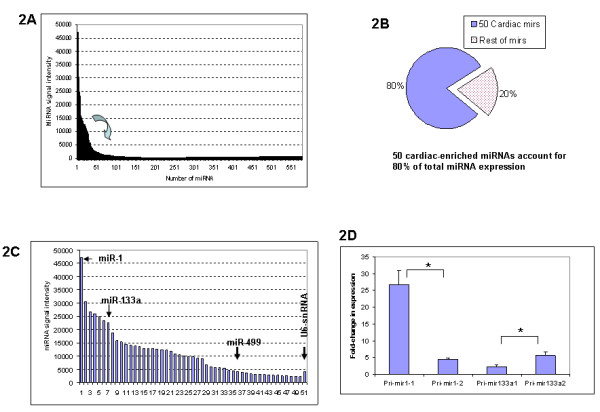
**The expression of microRNA in wild-type mouse heart**. **2A**. Approximately 50 microRNAs are cardiac-enriched microRNAs that are highly expressed in the adult mouse heart. **2B**. The 50 cardiac-enriched microRNAs account for approximately 80% of the total microRNA expression in the mouse heart, whereas the remaining 528 miRNAs account for only 20% of the total microRNA expression. **2C**. The expression levels of 50 cardiac-enriched microRNAs and U6-snRNA (reference gene). miR-1 ranks number 1 in expression, miR-133a ranks number 7 in expression. The expression level of miR-499 is similar to that of U6-snRNA. **2D**. Differential expression of pri-miRNA transcripts. Both pri-mir-1-1 and pri-mir-1-2 are processed into mature miR-1, but pri-mir-1-1 transcript level is 6-fold higher than that of pri-mir-1-2 (n = 3, p < 0.05*). The pri-mir-133a1 level is lower than that of pri-mir-133a2 (n = 3, p < 0.05*). These data suggest that the contribution of each pri-miRNA to the mature miRNA pool may be different.

To get an overview of microRNA expression in the mouse heart, the Exiqon microRNA array that detects 578 microRNAs was employed for the microarray analysis. The signal intensity of miRNAs in the wild-type mouse ranged from a low of 40 to a high of 47,243, with approximately 50 miRNAs having signal density above 2000 (Figure 2A). In terms of signal intensity, these 50 microRNAs accounted for approximately 80% of all the combined microRNAs expressed in the heart (Figure 2B and Table 1), indicating that they are cardiac-enriched miRNAs. When compared with U6-snRNA gene, which is a gene that was used as a reference, approximately 35 miRNAs were expressed higher than that of U6-snRNA genes (Figure 2C). The miR-1 was ranked number one in the level of expression among all the microRNAs detected, and it alone accounted for 7% of all the microRNA expression signals, and 9% of the 50 cardiac-enriched microRNA signals. Another important miRNA, mir-133a, was ranked number seven in terms of level of expression.

**Table 1:** The 50 cardiac-enriched microRNAs that are expressed in the wild-type adult mouse heart.

miR-1	miR-26b	miR-24	miR-144	let-7b
let-7f	miR-30a	miR-23a	miR-27a	miR-30d
miR-133b	miR-23b	miR-26a	miR-30e	miR-130a
miR-451	miR-29a	miR-16	miR-302d*	miR-923
miR-126	miR-30c	let-7d	miR-805	miR-15a
miR-22	miR-125b-5p	miR-690	miR-499	miR-486
miR-133a	let-7c	miR-709	miR-27b	miR-195
miR-30b	miR-143	let-7a	miR-29c	miR-101a
miR-208a	let-7g	let-7e	let-7i	miR-142-3p
miR-720	miR-378	miR-29b	let-7i	miR-126-5p

Although the majority of the miRNAs are transcribed from a single gene, some miRNAs are transcribed from more than one gene. For examples, the mature miR-1 is processed from pri-mir-1-1 and pri-mir-1-2 transcripts that are transcribed from two genes, mir-1-1 (on chromosome 2) and mir-1-2 (on chromosome 18), respectively. Bioinformatics analysis revealed that the 5-kb promoter sequence of these two genes was divergent, and that each gene promoter contains a different number of CArG-like sequences. To test the hypothesis that mir-1-1 and mir-1-2 genes may have different transcriptional activity, the pri-mir-1-1 and pri-mir-1-2 transcript level were measured with real-time RT-PCR. As shown in Figure 2D, pri-mir-1-1 level was 6-fold higher than that of pri-mir-1-2 (n = 3, p < 0.05). Similarly, the mature miR-133a is derived from both mir-133a1 gene (on chromosome 18) and mir-133a2 gene (on chromosome 2). Real-time RT-PCR showed that pri-mir-133a1 level was 2-fold lower than that of pri-mir-133a2 (n = 3, p < 0.05) (Figure 2D). These data suggest that the contribution of each pri-miRNA to the mature miRNA pool may be different.

### 2. Cardiac SRF level affects the expression of microRNAs in the mouse heart

**Figure 3:**
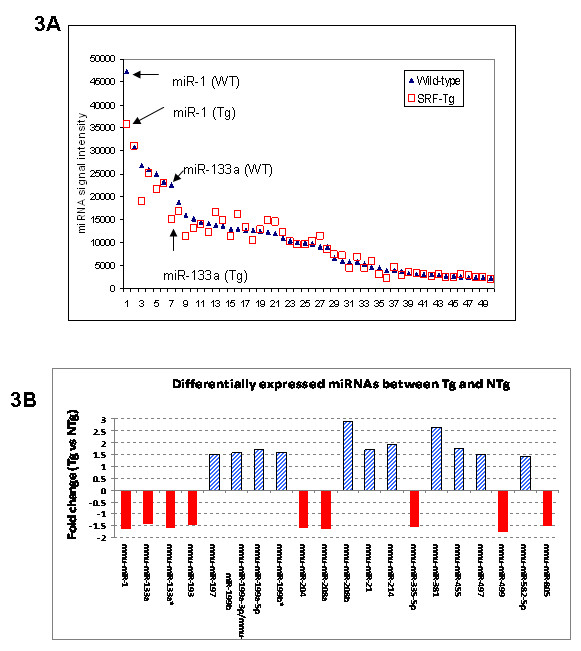
**The impact of SRF overexpression on the expression of cardiac miRNAs**. **3A**. Comparison of the expression of 50 cardiac-enriched miRNAs in adult SRF-Tg with that in age-matched wild-type mice. **3B**. The 20 microRNAs that are found to have at least 1-fold change in the hearts of adult SRF-Tg mice compared to that of age-matched wild-type (WT) mice. Both guide strand and passenger strand (*) of mir-133a are decreased in SRF-Tg vs. wild-type mice.

SRF is known to regulate mir-1, which regulates certain critical cardiac regulatory proteins that control the balance between differentiation and proliferation during cardiogenesis [4]. To identify other miRNAs that may be regulated by SRF, we examined the expression of cardiac miRNAs in response to SRF overexpression in the mouse heart. As shown in Figure 3A, SRF overexpression changed the expression of cardiac-enriched microRNAs to different extents.

**Figure 4:**
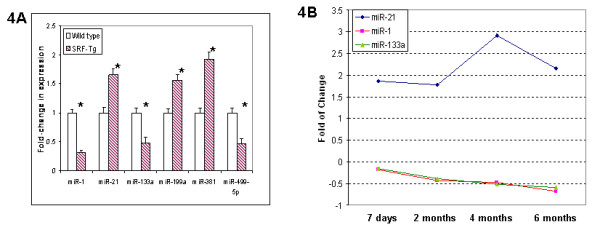
**SRF overexpression affected the expression of cardiac miRNAs**. **4A**. The expression of 6 miRNAs in adult wild-type vs SRF-Tg hearts was confirmed by real-time PCR. The data showed that the miRNA levels in SRF-Tg were altered significantly compared to that of wild-type mice (p < 0.05*, n = 3). **4B**. Altered expression of microRNAs occurred at as early as 7 days of age, and continued to 6 months of age in SRF Tg mice. miR-21 was increased in the heart of SRF Tg mice, but both miR-1 and miR-133a were decreased in the heart of SRF Tg mice compared to age-matched wild-type mice. The down-regulation of miR-1 correlates closely with that of miR-133a in SRF-Tg at various time points from 7 days to 6 months of age (p < 0.05, n = 3 for all time points, except n = 6 for miR-21 at 6 months).

Twenty microRNAs were significantly up or down-regulated by at least 1-fold in SRF-Tg versus wild-type mice (Figure 3B). Real-time RT-PCR analysis of 6 microRNAs confirmed the microRNA array data (Figure 4A).

We had previously observed that mild-overexpression of SRF resulted in the onset of cardiac hypertrophy at 6 months of age [12]. To define whether altered expression of microRNA might be the result of SRF overexpression, or a secondary effect caused by cardiac hypertrophy in the SRF-Tg heart, we determined miRNA expression at the ages of 7 days, 2 months and 4 months, and after the onset of the hypertrophic phenotype, at around 6 month of age. As shown in Figure 4B, altered expression of miRNA occurred at as early as 7 days after birth, long before the onset of cardiac hypertrophy and continued through 6 months of age, suggesting that SRF overexpression is likely the main factor contributing to dysregulation of microRNAs.

To test the hypothesis that a mild reduction of cardiac SRF level may result in the expression of microRNAs in the opposite direction to that which was observed in the SRF-Tg, we employed Anti-SRF-Tg, in which SRF protein expression was mildly decreased in the heart (Figure 1C). Reduced SRF protein in the heart resulted in decreased expression of ANF, increased expression of cardiac α-actin, MLC2v and SERCA2 expression (Figure 1D); it also resulted in improved cardiac performance in response to stress [17].

**Figure 5:**
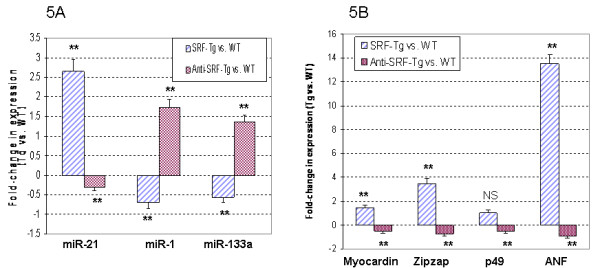
**Reduced SRF protein levels in mouse hearts resulted in gene expression patterns that were opposite to what was observed in SRF-Tg heart**. **5A**. The up-regulation of miR-21, and the down-regulation of miR-1 and miR-133a were observed in SRF-Tg compared to wild-type (WT) mouse heart (P < 0.01**, n = 3). Interestingly, the down-regulation of miR-21, but up-regulation of miR-1 and mir-133a were observed in Anti-SRF-Tg compared to wild-type mouse heart (p < 0.01**, n = 3). **5B**. Myocardin, Zipzap, p49/Strap and ANF were up-regulated in the SRF-Tg compared with the wild-type mouse heart, but down-regulated in the Anti-SRF-Tg compared with the wild-type mouse heart (p < 0.01**, n = 3).

Real-time RT-PCR analysis revealed that mildly reduced SRF resulted in the down-regulation of miR-21 expression, but up-regulation of both miR-1 and miR-133a (Figure 5A). These results were in the opposite direction compared with that which was observed with mild SRF overexpression in the SRF-Tg heart.

We further tested the expression of three SRF cofactors, namely myocardin, p49/Strap and Zipzap in these two mouse models, and used ANF as a reference. As shown in Figure 5B, increased expression of myocardin, Zipzap and ANF was observed in SRF-Tg, while decreased expression of myocardin, Zipzap and ANF was observed in Anti-SRF transgenic mouse heart compared to their age-matched wild type mice, respectively (p < 0.05, n = 3). There was no significant change in the expression of p49/Strap in SRF-Tg versus wild-type heart (NS, n = 3), but p49/Strap was decreased in the anti-SRF-Tg versus wild-type heart (p < 0.01, n = 3).

### 3. SRF regulates the transcription of pri-miRNA transcript

**Figure 6:**
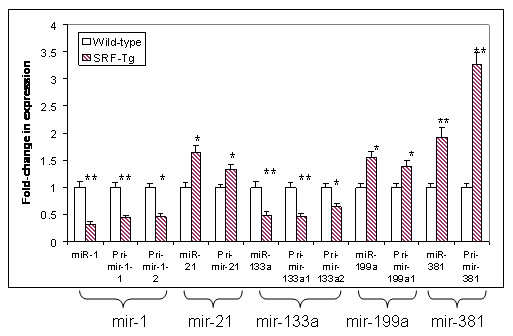
**The expression of mature miRNA (miR) and pri-miRNA transcript(s) in adult SRF-Tg compared with that in age-matched wild-type mice**. The expression of miR correlates with its corresponding pri-microRNA transcript(s), indicating SRF regulates pri-microRNA transcription. The change in expression of the microRNAs between SRF-Tg and wild-type mice was significant (p < 0.05*, or p < 0.01**, n = 3).

The transcription of pri-miRNA is the first step in miRNA biogenesis [26]. Pri-miRNA is then cleaved into pre-miRNA before maturity [27]. To define whether increased SRF protein in the heart could affect pri-miRNA transcription, the pri-miRNA expression level in SRF-transgenic mouse heart was compared with that in the wild-type. Interestingly, all the pri-miRNAs examined were altered in SRF-Tg compared to wild-type mouse, suggesting that SRF affects pri-miRNA transcription (Figure 6).

To determine whether pri-miRNA level correlates with mature miRNA level, the pri-miRNA levels were compared with that of their corresponding mature forms (miR). As shown in Figure 6, when pri-mir-1-1 and pri-mir-1-2 transcripts were down-regulated, so was miR-1 mature form; when pri-mir-133a1 and pri-mir-133a2 transcripts were down-regulated, the same was true for miR-133a mature form. Similarly, when pri-mir-21, pri-mir-199a, and pri-mir-381 primary transcripts were up-regulated, their mature miRNA forms were up-regulated as well (Figure 6). These data suggest that SRF regulates pri-miRNA expression, thereby affecting the mature miRNA level.

### 4. SRF regulates mir-21 expression

MiR-21 was increased in SRF-Tg heart (Figure 4A, B, 5A) compared to wild-type mice. However, miR-21 was decreased in Anti-SRF-Tg heart compared to that of the wild-type mouse heart (Figure 5A), indicating that miR-21 expression is regulated by the SRF protein. To evaluate the mir-21 gene promoter region, the DNA sequences of mouse, rat and human mir-21 genes and flanking regions were obtained from Genbank database. The mir-21 pri-miRNA sequence was obtained from GenBank sequence AY699265 and also from the sequence reported by Fujita et al. [28].

**Figure 7:**
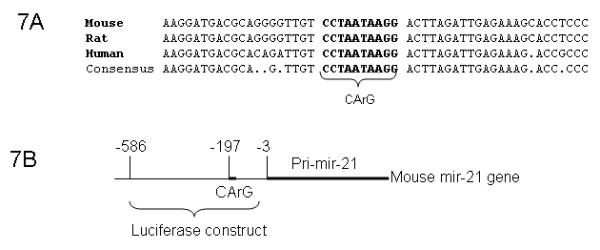
**The promoter region of the mir-21 gene**. **7A**. The DNA sequence of mir-21 promoter region was conserved among mouse, rat and human, and a consensus CArG motif was found in the promoter of all three species. **7B**. The mouse mir-21 gene sequence corresponding to pri-mir-21 and its promoter region. A CArG motif is 197 bp upstream of mir-21 primary transcript. A pGL3-luciferase construct containing promoter fragment (-586 bp to -3 bp) was generated and used in the present experiment (see details in Materials and Methods).

Analysis of the mir-21 promoter region revealed that the mir-21 gene has a classic SRF binding site or CArG element that is composed of "CCTAATAAGG", which is found to be 197 bp upstream of the pri-mir-21 transcript start point (Figure 7A and 7B). The human, rat and mouse mir-21 promoter region is relatively conserved, as is the classic SRF binding site (Figure 7A).

**Figure 8:**
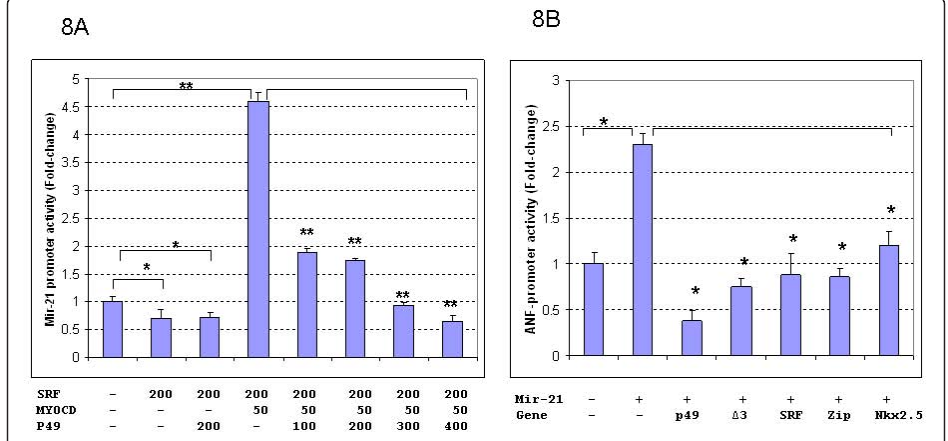
**Mir-21 and ANF Promoter activity assays**. **8A**. Mir-21 promoter is regulated by SRF and its cofactors, myocardin and p49/Strap. In vitro transfection assay indicates that both SRF and p49/Strap repressed the mir-21-luciferase reporter activity, respectively (p < 0.05, n = 3). Myocardin (MYOCD) strongly activated the mir-21 promoter activity (p < 0.05). p49 also repressed mir-21-luciferase reporter activity induced by myocardin in a dose-dependent manner (p < 0.01 **, n = 3). **8B**. The mir-21 microRNA up-regulates ANF expression. The microRNA mir-21 up-regulated ANF gene promoter (p < 0.05*, n = 3). P49/Strap (p49), SRF isoform Δ3, SRF, Zipzap (zip) and Nkx2.5 all down-regulated the ANF gene promoter activity that was induced by mir-21 microRNA (p < 0.05, n = 3).

To test whether the mir-21 promoter would respond to the regulation of SRF and SRF cofactors in vitro, a mir-21 promoter-luciferase construct was generated, which contains a 0.58 kb DNA fragment of mir-21 promoter (Figure 7B). Transfection assay using SRF, p49/Strap and myocardin was performed. As shown in Figure 8A, the mir-21 gene promoter was repressed by SRF and p49/Strap alone. The mir-21 promoter was strongly activated by myocardin, but the activation induced by myocardin was repressed by p49/Strap at various concentrations (Figure 8A).

**Figure 9:**
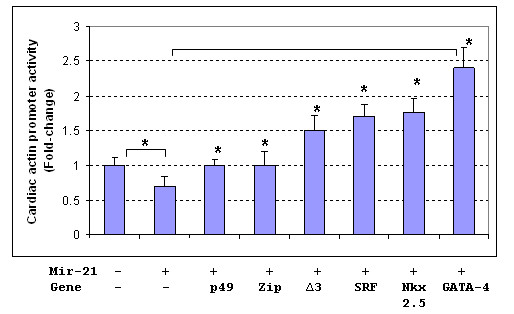
**The mir-21 microRNA represses cardiac actin expression, but SRF and its cofactors activate cardiac actin expression**. The mir-21 microRNA repressed cardiac actin gene expression compared with control (P < 0.05*, n = 3). Transfection of several other genes up-regulated the cardiac actin gene compared with mir-21 alone: p49/Strap (p49), Zipzap (Zip), SRF-Δ3 isoform (Δ3), SRF, Nkx2.5, GATA-4, respectively (P < 0.05*, n = 3).

To examine the effect of increased miR-21 microRNA level on cardiac gene expression, a mir-21 expression vector containing mir-21 DNA sequence was generated using pSilencer4.1 vector. The pSilencer4.1-mir-21 expression vector was cotransfected with ANF promoter and cardiac actin promoter plasmid vectors, respectively. As shown in Figures 8B and Figure 9, miR-21 expression upregulated ANF promoter activity, while it repressed cardiac actin expression. The SRF cofactors p49/Strap, Zipzap, Nkx2.5, GATA-4 had modulatory effects on both ANF and cardiac actin expression (Figures 8B and 9).

## Discussion

The present study has several findings. We observed a broad range of miRNAs that are expressed in the wild-type mouse heart. Cardiac-specific overexpression of SRF significantly impacted a number of miRNAs, which are potential SRF target miRNAs. Future investigation of these miRNAs may enhance our understanding of their role in the SRF-mediated regulation of cardiac development, maturation and aging. Interestingly, we found that SRF regulates microRNA biogenesis, specifically at the stage of transcription of the pri-miRNA transcript. The expression levels of miR-1, miR-133a and miR-21 were observed to be in the opposite direction with reduced cardiac SRF level in the Anti-SRF-Tg mouse. Our study reports for the first time a successful attempt to reverse the expression of these three miRNAs, which are frequently dysregulated in cardiac diseases, through targeting their upstream regulator.

It is known that miRNA expression is altered in a number of diseases and pathological conditions, including cardiac hypertrophy and heart failure [3,13,14,29,30]. The miRNA profile has been shown to serve as an impressive phenotypic signature. Therefore, miRNA has the potential to be developed as diagnostic and/or prognostic tools [31]. On the other hand, since individual miRNAs are dysregulated either negatively or positively with disease, strategies are needed to counteract or reverse the altered expression, potentially through reconstituting and/or targeting regimens, respectively [32-34]. A deeper understanding of miRNA biogenesis, as well as the regulation of each step in the miRNA maturation process and the involvement of signaling pathways and transcription factors is needed.

**Figure 10:**
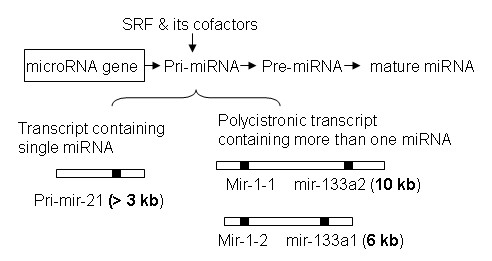
**A model of miRNA regulation mediated by SRF and its cofactors, including myocardin, p49/Strap, Zipzap and other proteins**. Generally, the pri-miRNA transcript contains one miRNA (e.g pri-mir-21), but it can also contain more than one miRNAs (e.g. mir-1 and mir-133a). SRF regulates the transcription activity of both types of pri-miRNAs, thereby affecting the downstream mature miRNA level. The length of three representative primary transcripts: pri-mir-21 is over 3 kb, "pri-mir-1-1 and pri-mir-133a2" is 10 kb, and "pri-mir-1-2 and pri-mir-133a1" is 6 kb.

It has been reported that miRNAs undergo multiple steps during biogenesis, in which three forms of miRNAs are produced: pri-miRNA, pre-miRNA and mature miRNA [1,35]. Multiple proteins are involved in this process. RNA polymerase II or RNA polymerase III transcribes the transcripts of pri-miRNA; a microprocessor complex cleaves pri-miRNA to generate pre-miRNA; and another endonuclease, Dicer, cleaves the pre-miRNA to release a miRNA-miRNA duplex. The miRNA strand is then stripped away from the duplex to leave a mature, approximately 22-nucleotide miRNA [36]. These individual steps can be targeted to increase or decrease the miRNA level. Our data reveal that SRF executes it regulatory role in miRNA biogenesis through transcriptional regulation where it controls the amount of pri-miRNA available for downstream miRNA maturation process. We have proposed a model of miRNA regulation by SRF and its cofactors (Figure 10).

Mir-21 is known to be up-regulated in many forms of cancer as well as in the heart during cardiac hypertrophic growth and heart failure [37-40]. In the failing heart, miR-21 levels are increased in fibroblasts where it inhibits sprouty homologue 1 (Spry1) and promotes fibrosis [16]. In vivo, the silencing of miR-21 by a specific "antagomir" in a mouse pressure-overload-induced disease model was reported to reduce cardiac ERK-MAP kinase activity, inhibit interstitial fibrosis and attenuate cardiac dysfunction [16]. It has been reported that TGF-β and BMP signaling promote a rapid increase in the expression of mature miR-21 at the post-transcriptional level through accelerating the process of pri-miR-21 into precursor miR-21 (pre-miR-21) by the DROSHA complex [41]. Mir-21 is one of the miRNAs that was significantly increased in the SRF-Tg heart, but was decreased in the anti-SRF-Tg heart. However, the mir-21 gene promoter was repressed by SRF and p49/Strap in transfection assay, and the promoter was strongly induced by myocardin. Similarly, we observed that SRF activates both cardiac α-actin and MLC-2v promoter-luciferase DNA constructs in vitro assay, but SRF repressed the expression of both mRNA in the mouse heart [18,22]. These data indicate that the regulation of mir-21 gene expression is complex, and involves SRF in coordination with multiple SRF cofactors, including myocardin, p49/Strap and Zipzap. The fact that a "knock-down" of SRF level could decrease the mir-21 level provides an opportunity for future studies manipulating mir-21 level through the SRF-mediated signaling pathway.

Both miR-1 and miR-133a are produced from the same polycistronic transcripts, which are encoded by two separate genes in the mouse and the human genomes [42]. The mouse pri-mir-1-1 and pri-mir-133a-2 are transcribed into a polycistronic transcript that is 10 kb in length, and the pri-mir-1-2 and pri-mir-133a-1 are transcribed into another polycistronic transcript that is 6 kb in length [42]. Our data revealed that the down-regulation of miR-1 correlates closely with that of miR-133a in the SRF-Tg at various time points from 7 days to 6 months of age (Figure 7B). These findings suggest that SRF may regulate these two miRNAs at the level of polycistronic transcription, rather than at each individual miRNA (pri-mir-1 or pri-mir-133a) transcription, thereby keeping the expression of both miRNAs closely correlated. Since mir-1-1 and mir-1-2 genes are located on two different chromosomes, their expression is divergent. The pri-mir-1-1 is expressed at 6-fold higher than pri-mir-1-2. Therefore, the contribution of pri-mir-1-1 to the mature miR-1 pool may be greater than that of pri-mir-1-2. Given the fact that targeted mutation of mir-1-2 gene resulted in embryonic myocardial dysfunction and half of the mutant mice suffered early death due to ventricular septal defect (VSD) [4], one might speculate that a targeted mutation of mir-1-1 gene would also cause equally (or more) severe consequences.

The miR-1 is the most abundant miRNA that is expressed in the heart. Our present study revealed that miR-1 accounted for 7% of all the 578 miRNAs detected by the microarray. Mir-1 and mir-133a are down-regulated in cardiac hypertrophy and cardiac failure, suggesting that they may play a role in the underlying pathogenesis [14,43]. It is plausible that increasing mir-1 and mir-133a level at a specific time point may have potentially beneficial effects against the pathological conditions. Matkovich et al reported that an increase of mir-133a level in the postnatal heart has beneficial effects against cardiac fibrosis after transverse aortic constriction [44].

In conclusion, our current study demonstrates that cardiac-specific overexpression of SRF leads to altered expression of cardiac miRNAs, especially the down-regulation of miR-1 and miR-133a, and up-regulation of miR-21, the dysregulation of which is known to contribute to cardiac hypertrophy. We observed that SRF plays a role in the regulation of miRNA biogenesis, specifically at the level of transcription of pri-miRNA. Reducing cardiac SRF level using the antisense-SRF transgenic approach led to the expression of miR-1, miR-133a and miR-21 in the opposite direction to that of SRF overexpression. Our findings may help in the future development of therapeutic interventions through targeting of the SRF-mediated signaling pathways.

## Competing interests

The authors declare that they have no competing interests.

## Authors' contributions

XMZ participated in the design of the study, carried out part of the experiments and drafted the manuscript. GA participated in the coordination of the study and the writing of the manuscript. SH participated in bioinformatics analysis. JYW led the study and participated in the design of the study, the statistical analysis and overall interpretation of results. All authors read and approved the final manuscript.
